# Impact of temperature and humidity on SARS-CoV-2 transmissibility: a systematic review and meta-analysis

**DOI:** 10.3389/fpubh.2025.1570002

**Published:** 2025-10-10

**Authors:** William Kerobe, Abrahaman Said Msellem, Paul Alikado Sabuni, Farida Iddy Mkassy, Peter Martin Chilipweli, Anthony Kapesa, Benson R. Kidenya, Philip Ayieko, Deogratius Bintabara, Eveline T. Konje

**Affiliations:** ^1^Department of Epidemiology and Biostatistics, Catholic University of Health and Allied Sciences, Mwanza, Tanzania; ^2^Department of Community Medicine, Catholic University of Health and Allied Sciences, Mwanza, Tanzania; ^3^Department of Biochemistry and Molecular Biology, Catholic University of Health and Allied Sciences, Mwanza, Tanzania; ^4^Department of Infectious Disease Epidemiology and International Health, London School of Hygiene and Tropical Medicine, London, United Kingdom; ^5^Mwanza Intervention Trials Unit, Mwanza, Tanzania; ^6^Department of Epidemiology and Biostatics, University of Dodoma, Dodoma, Tanzania

**Keywords:** SARS-CoV-2, COVID-19, climatic factors, temperature, humidity, transmissibility

## Abstract

**Background:**

The SARS-CoV-2 pandemic remains crucial for understanding the epidemiology of future respiratory infections. Gaining insights into the climatic factors influencing the transmissibility of SARS-CoV-2 is an important public health issue in the control and prevention of the disease. Hence, this study aimed to assess the association between SARS-CoV-2 transmissibility and both humidity and temperature.

**Methods:**

Articles published between December 2019 and August 2024 were identified from PubMed, Africa Journal Online, Science Direct, and Hinari databases following PRISMA guidelines. The focus was on studies that reported transmissibility based on basic reproductive number, specifically correlation coefficients between basic reproductive number and temperature, or humidity, or corresponding regression coefficients, and their standard errors. The Joanna Briggs Institute (JBI) Critical Appraisal Checklist was used to assess the risk of bias. Random effect models were applied. The meta-analysis was done in R version 4.3.0.

**Results:**

9 studies qualified, but 5 were excluded for missing coefficients, leaving 4 for meta-analysis. The study analysis revealed a significant negative correlation between temperature and SARS-CoV-2 transmissibility (r = −0.509, 95% CI: −0.680 to −0.338, *p* < 0.001). Similarly, a significant but weaker negative correlation was found between humidity and SARS-CoV-2 transmissibility (r = −0.426, 95% CI: −0.548 to −0.303, *p* < 0.001). A unit increase in humidity measured in percentage was associated with a decrease in transmissibility by 0.006 (95% CI: −0.007 to −0.004, *p* < 0.001), while a unit increase in temperature in Celsius (°C) was associated with a reduction of transmissibility by 0.008 (95% CI: −0.030 to −0.030, *p* < 0.001).

**Conclusion:**

Temperature and humidity were negatively associated with SARS-CoV-2 transmissibility; thus, disease transmissibility decreased as temperature or humidity increased. Climatic factors are important considerations for effective disease surveillance and preparedness strategies.

**Systematic review registration:**

https://www.crd.york.ac.uk/PROSPERO, CRD42025637440.

## 1 Introduction

In December 2019, the world experienced the devastating impact of SARS-CoV-2, a deadly virus that originated in Wuhan, China, and quickly spread worldwide (1). In March 2020, the World Health Organization (WHO) declared SARS-CoV-2 a pandemic and a global public health emergency (2). To date, the disease has caused more than 7 million deaths and has impacted many others through job losses, and severe health complications (3, 4). A globally coordinated effort that accelerated research, vaccine distribution, enhanced surveillance systems, and ensured equitable access to diagnostics and treatments helped in the pandemic control. The WHO declared on May 5, 2023, that SARS-CoV-2 infection was no longer considered a public health emergency (5). However, the disparities in terms of transmission, incidence rate, morbidity, and mortality due to SARS-CoV-2 have been reported across different regions and continents (4). As of April 2024, more than 705 million cases and 7 million deaths have occurred globally. Europe recorded more than 253 million cases and 2.1 million deaths, while Asia reported more than 222 million cases and 1.55 million deaths. North America reported more than 132 million cases and nearly 1.7 million deaths, compared to South America’s 70 million cases and 1.37 million deaths (4). Oceania remains relatively spared, with more than 14.9 million cases and 33,015 deaths, and Africa has approximately 12.9 million cases with about 259,000 deaths (4). This highlights the need to thoroughly learn and investigate the determinants as well as the diversity of transmissibility of SARS-CoV-2 infection to better manage similar pandemics in the future.

The underlying causes of discrepancies in SARS-CoV-2 outcomes remain unclear. There are suggestions in literature that geographic differences in viral mutations drive regional variations in transmission and fatality rates, but evidence from other studies indicates these differences are primarily due to demographic factors and public health responses (6, 7). Separately, the underreporting and concealing of SARS-CoV-2 cases, coupled with limited testing options in some geographic regions, could contribute to the discrepancies (8). Social factors like social interaction and extensive travel, along with geographical and climatic factors, might also have played a role in the transmission of SARS-CoV-2 (9–11). Furthermore, research suggests that climatic factors, including temperature, humidity, wind speed, rainfall, and even gravity, may influence the emergence and spread of SARS-CoV-2 (12). Focusing on temperature and humidity is crucial, as studies have reported a possible positive correlation between these factors and the transmissibility and incidence of SARS-CoV-2 (13, 14). In contrast, other studies have found a negative correlation between low temperature and SARS-CoV-2 transmissibility (11, 15), suggesting that there may be confounding factors that distort the relationship. These seemingly conflicting results underscore the complexity of climate on the transmissibility of SARS-CoV-2 and highlight the need for further, more nuanced investigation of the influence of climatic factors during the pandemic. While air quality, wind speed, gravity, and rainfall are significant meteorological factors in SARS-CoV-2 transmission by enhancing virus stability and weakening host defenses (15) there are a limited number of studies exploring their relationship with the transmissibility of SARS-CoV-2.

To understand the inherent ability of SARS-CoV-2 to spread from one host to another under the influence of temperature and humidity, the literature suggests the use of reproduction numbers (16–19). This is because other methods, such as case counts, can be affected by reporting delay and underreporting, which differ by location, and are challenging to manage (16–18). In contrast, the basic reproduction number (R0) provides a direct measure of SARS-CoV-2 transmissibility by quantifying the average number of infections caused by one infection in the population before any public health measure was put into effect (16–19). Ideally, R0 assumes a fully susceptible population, unchanging environmental conditions, consistent human interactions, and no medical interventions that could alter transmission dynamics. Thus, the estimated R0 can be used as a reliable indicator of SARS-CoV-2 transmissibility compared to daily case counts. From this perspective, employing R0 enhances the study by providing a comprehensive understanding of the transmissibility of SARS-CoV-2 about temperature and humidity on a global scale. Hence, this review aims to update and further evaluate available literature on the correlation between temperature/humidity and SARS-CoV-2 transmissibility, ultimately providing useful insights for incorporating climatic factors into surveillance and early warning strategies for future outbreaks.

## 2 Methods and materials

### 2.1 Eligibility criteria

This systematic review considered studies that estimated and reported the effects of temperature and humidity on the transmissibility of SARS-CoV-2 infection, with a focus on reproduction number (R0). The inclusion criteria were all peer-reviewed articles written in English examining the relationship between climatic factors (i.e., humidity and temperature) and transmissibility of SARS-CoV-2. Studies with insufficient data or unclear methodology, experimental studies, reviews, and duplicate publications were excluded. Primary studies (i.e., original articles) were retrieved from PubMed, Africa Journal Online, Science Direct, and Hinari databases following PRISMA guidelines. In addition, the search strategy was defined based on the population, exposure, and outcome, i.e., (PEO). Specifically, observational studies that involved populations of humans diagnosed with SARS-CoV-2, with humidity and temperature being climatic exposures, and transmissibility of SARS-CoV-2 based on reproduction number being an outcome of interest, were considered for inclusion.

### 2.2 Screening procedure

The retrieved articles were processed and managed using Rayyan, an AI-powered systematic review management platform that automatically handles duplication. Rayyan provides an interactive interface to assist researchers in organizing, managing, and accelerating literature reviews (20). Three reviewers (PAS, WKA, and ASM) independently and blindly screened the titles and abstracts. In cases of disagreement or uncertainty during the screening process, a fourth reviewer (ETK) was consulted to resolve conflicts and determine article inclusion. During full-text screening, four reviewers (PAS, WKA, ASM, and ETK) worked in pairs to assess each article, ensuring adherence to the inclusion and exclusion criteria and assessing its relevance as a data source for this study.

### 2.3 Data extraction

A standardized data extraction form was developed based on the Cochrane Consumers and Communication Review Groups (21) to extract important data from each included study. Four reviewers (PAS, WKA, ASM, and ETK) in pairs extracted data using the following characteristics: author, year, study duration, design, sample size, correlation coefficient, regression coefficient, and standard errors. The correlation coefficient or regression coefficient was based on the relationship between temperature or humidity and R0.

### 2.4 Risk of bias assessment

All the studies included in the analysis were rigorously assessed using the Joanna Briggs Institute (JBI) Critical Appraisal Checklist tool (22). The checklist for cross-sectional studies consists of eight criteria, which include the description of study subjects and the setting, measurement of exposure, objective criteria for measuring the condition, identification of confounding factors, strategies to address confounding factors, measurement of outcomes, and the statistical analysis used. Each component was rated as “yes,” “no,” “unclear,” or “not applicable.” A high risk of bias classification corresponds to 0–3 “yes” scores, moderate risk corresponds to 4–6 “yes” scores, and low risk corresponds to 7–8 “yes” scores. We worked independently to assess the quality of each study, and any disagreements were resolved through discussion within the review team (Table 1).

**Table 1:** Risk bias assessment based on JBI Critical Appraisal Checklist for analytical cross-sectional studies.

S/N	Papers	Igor et al.	Smith et al.	Guo et al.	Wang et al.	Si et al.	Kong et al.	Landier et al.	Ogunjo et al.	Pahuja et al.
1	Were the criteria for inclusion in the sample clearly defined?	Yes	Yes	Yes	Yes	Yes	Yes	Yes	Yes	Yes
2	Were the study subjects and the setting described in detail?	Yes	Yes	Yes	Yes	Yes	No	No	Yes	Yes
3	Was the exposure measured in a valid and reliable way?	Yes	Yes	Yes	Yes	Yes	No	No	Yes	Yes
4	Were objective, standard criteria used for measurement of the condition?	Yes	Yes	Yes	Yes	Yes	No	Yes	Yes	Yes
5	Were confounding factors identified?	Yes	Yes	No	No	No	No	No	Yes	Yes
6	Were strategies to deal with confounding factors stated?	No	Yes	No	No	No	No	No	Yes	Yes
7	Were the outcomes measured in a valid and reliable way?	Yes	Yes	Yes	Yes	Yes	Yes	No	Yes	Yes
8	Was appropriate statistical analysis used?	Yes	Yes	Yes	Yes	Yes	Yes	Yes	Yes	Yes
	*Risk of bias*	*Low*	*Low*	*Low*	*Moderate*	*Moderate*	*High*	*High*	*Low*	*Low*

Scale 7–8 “yes” = Low risk; 4–6 “yes” = Moderate risk; 0–3 “yes” = High risk. JBI, Joanna Briggs Institute, a Critical Appraisal Checklist tool for risk bias assessment.

### 2.5 Data analysis

The data obtained from the primary studies were first synthesized narratively to present the common observed findings from reviewed articles, aiming to understand the relationship between temperature, humidity, and the transmissibility of SARS-CoV-2. For the studies with sufficient quantitative data, two separate meta-analyses were conducted to obtain the pooled effect of: (i) temperature on SARS-CoV-2 transmissibility and (ii) humidity on SARS-CoV-2 transmissibility. Further, within each meta-analysis two effect size types (correlation coefficient and regression coefficient) were used to calculate a pooled effect according to the type of metric used to quantify the association between temperature or humidity and SARS-CoV-2 transmissibility in the primary study. Heterogeneity was assessed by inspecting forest plots, and was quantified by calculating the Q-statistic, *p*-value, I^2^ and prediction interval. To enhance the efficiency of the estimate, both fixed-effect and random-effects models were applied to estimate pooled effect sizes, allowing comparison under assumptions of homogeneity and heterogeneity across studies (23). Thus, whether the true effect size is shared across all studies (for fixed or common effect model) or varies among studies due to methodological and contextual differences (for random model) (21). All the statistical computation and estimates was done in R software version 4.3.0 at 5% significance level (24).

## 3 Results

### 3.1 Study selection

**Figure 1:**
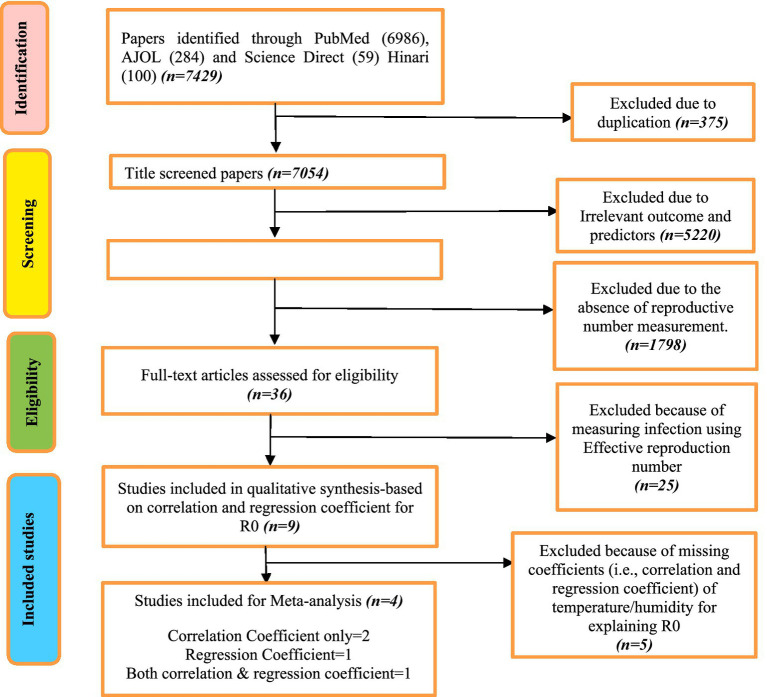
PRISMA flow chart to show different phases of the systematic review.

A total of 7,429 studies were identified and retrieved from PubMed (6986), AJOL (284), Science Direct (59), and Hinari (100). Of all the retrieved studies, 375 (5.0%) were excluded due to duplication. After title screening, 5,220 (74.0%) were excluded due to irrelevant outcomes and predictors. During the abstract screening, 1,798 (98.0%) were excluded because they lacked the measurement of the reproductive number to assess SARS-CoV-2 transmissibility. Finally, during full-text screening, 25 (69.4%) were excluded because they used the effective reproduction number as opposed to the basic reproductive number. This resulted in nine studies that qualified to be included in the systematic review. Four studies (25–28) reported correlation and/or regression coefficients for inclusion in meta-analysis (Figure 1). Two out of the four studies reported correlation coefficients only (25, 27), one reported both correlation and regression coefficients (26), and one study conducted across two countries reported only the regression coefficients separately for each country (28). Furthermore, in the risk of bias assessment, we found that most studies have a low risk of bias, meeting 7–8 criteria, indicating strong reliability and valid research practices. However, two studies showed a moderate risk, missing essential criteria like confounding factor management, which may affect their findings (28, 29). Notably, two studies exhibited a high risk of bias (30, 31), meeting only three or fewer criteria, raising concerns about the validity of their results due to methodological shortcomings such as inadequate descriptions of adjustments for confounding factors and unreliable measurements.

### 3.2 Study characteristics

Of all the nine included studies, research was conducted across different geographical regions and years. These studies span from 2020 to 2022, with the majority published in 2021. Geographically, some studies focus on global coverage (25, 30, 31). While other studies focus on country-specific analyses, including the USA (27, 28), China (26, 28, 29), Nigeria (32), and India (31). In addition, some studies were examining a single city, such as those conducted in China and India (29, 33). Larger studies encompass broader regional samples, including studies covering 100 cities in China and extensive investigations across 50 and 1,005 counties in the USA (25, 26). Global studies also differ in their sample size, with sizes ranging from 6 to 118 cases worldwide (28, 31). The study conducted in Nigeria focuses on 7 states, offering a national perspective within a specific regional context (32). All the studies provide perspective in understanding the association between climatic factors (humidity and temperature) on SARS-CoV-2 transmissibility (Table 2).

**Table 2:** Summary of the effect of temperature and humidity on transmissibility of SARS-CoV-2.

S/N	Author	Years	Zone/Country	Sample size (country/cities)	Correlation coefficient		Regression coefficient				Study design		Technique used	Key finding	
					Temperature	*p*-value	Humidity	*p*-value	Temperature	SE	Humidity	SE			
1	Igor et al.	2021	Global	118	−0.390	0.0120	−0.350	0.0039					Ecological	SEIR	There is negative correlation between Basic reproduction number and weather (temperature and humidity).
2	Smith et al.	2021	USA (counties)	50	−0.675	0.0045	−0.561	0.4600					Ecological	SEIR	UV radiation is a very weak predictor of R0. Temperature and absolute humidity show a sufficiently moderate correlation on R0.
3	Guo et al.	2020	China (Cities)	11	−0.459	0.0100	−0.391	0.0010	−0.010	0.0210	−0.004	0.0010	Ecological	SEIR	R0 negatively correlated with both temperature and humidity.
4	Wang et al.	2021	China (cities)	100					−0.026	0.0049	−0.0076	0.0011	Cross-sectional	Fama-MacBeth	Statistically significant inverse relationship between CoV-2 transmissibility and both temperature and relative humidity in both China and the USA.
			USA (Counties)	1,005					−0.020	0.0052	−0.008	0.0034			
5	Si et al.	2021	China (Cities)	1									Ecological	GAM/SEIR	An average lag of 0–8 days temperature after adjusted with Relative humidity was associated with a daily decrease in Risk of transmissibility in Wuhan city. Significant negative association between Temperature and reproductive number. Negative, insignificant association between relative humidity and reproductive number.
6	Landier et al.	2021	Global	6									Ecological	SEIR	SARS-CoV-2 transmission to weather/climate, with each 1 °C reduction in mean regional temperature below 10 °C leading to a 0.16-unit increase in R0, or each 1 g/m3 reduction in AH leading to a 0.15-unit increase in R0.
7	Kong et al.	2021	Global	58									Ecological	GAM	Temperature and humidity did not have strong relationships with R0 but were positive.
8	Ogunjo et al.	2022	Nigeria (State)	7									Ecological	SEIR	Temperature and humidity showed negative correlations in some locations.
9	Pahuja et al.	2021	India (Cities)	1									Ecological	SEIR	Increasing temperature decreases SARS-COV-2 infection. No significant correlation of humidity or wind speed on SARS-COV-2 infection.

SEIR, Susceptible Exposed Infected Recovery; GAM, Generalized additive model. Source: Reviews Data of SARS-COV-2 from 2019 to 2024.

### 3.3 Narrative study synthesis

The reviewed studies indicate an inverse relationship between temperature and humidity on the transmissibility of SARS-CoV-2. While a weak negative correlation was established between SARS-CoV-2 and both temperature and humidity (25, 26), Other studies reported a moderate correlation between temperature (r = −0.675) and humidity (r = −0.561) with SARS-CoV-2 transmissibility (27, 32). Similarly, a negative correlation between temperature and humidity on SARS-CoV-2 transmissibility was reported (30, 31). Evidence also indicates that a decrease in temperature and humidity contributes to increased transmissibility, especially in the transition from summer to winter (33). Overall, the reviewed evidence suggests that lower temperature and humidity are correlated with increased SARS-CoV-2 transmissibility, with correlation strengths ranging from weak to moderate across studies.

Regression analysis further supports the inverse association of humidity and temperature on SARS-CoV-2 transmission (28, 33). Studies conducted in China and the USA revealed negative correlations between temperature/relative humidity and R0 (28). This inverse relationship was also emphasized in other studies (26, 33), suggesting that rising temperature and humidity may reduce SARS-CoV-2 transmissibility. Also, findings indicate a weak relationship between relative humidity and SARS-CoV-2 transmissibility (29). Conversely, while other studies report a positive relationship between humidity and SARS-CoV-2 transmissibility, an inverse association between temperature and SARS-CoV-2 transmissibility was observed (34). Notably, the findings on the role of humidity in SARS-CoV-2 transmissibility remain inconsistent. One study reported a weak association between relative humidity and transmissibility (29), whereas another observed an inverse relationship (34). However, most of the studies consistently noted an inverse relationship between SARS-CoV-2 transmissibility on temperature (34).

### 3.4 Meta-analysis results

**Figure 2:**
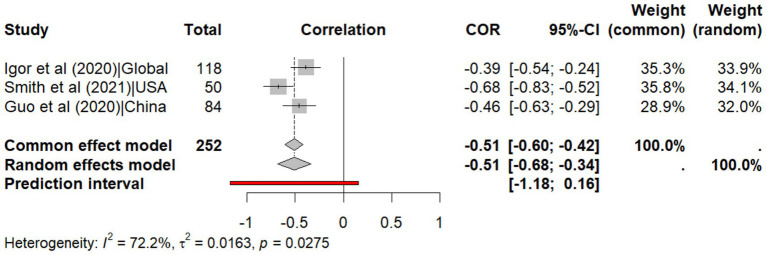
Meta-analysis of the correlation coefficients for temperature on explaining the transmissibility of SARS-CoV-2.

**Figure 3:**
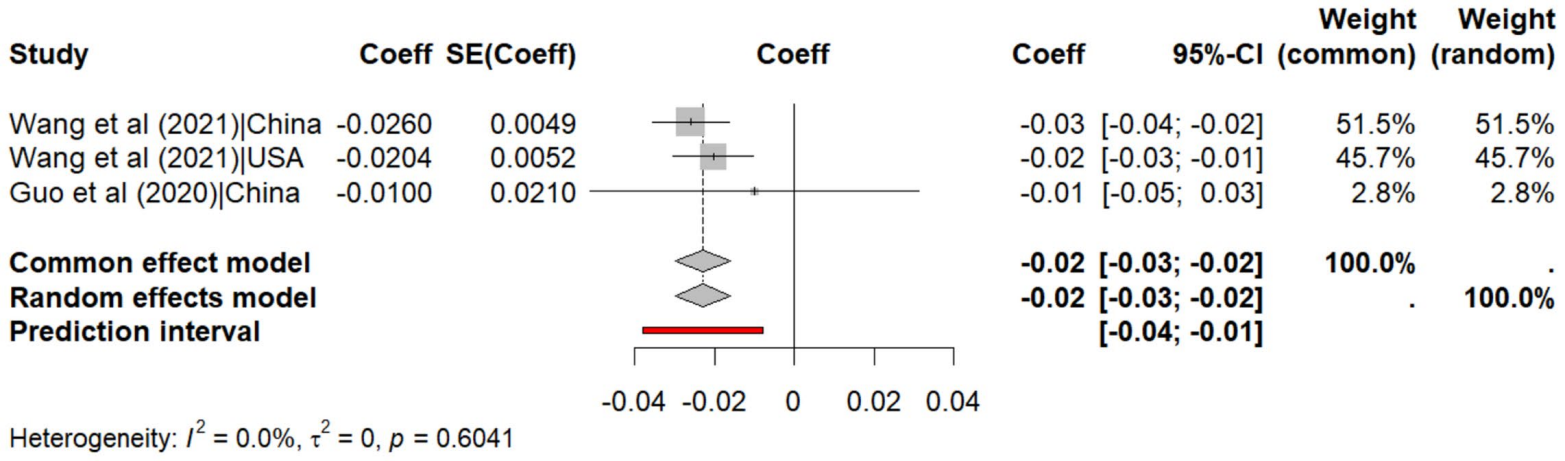
Meta-analysis of the effect size of regression coefficients for temperature on explaining the transmissibility of SARS-CoV-2.

The meta-analysis of correlation coefficients indicates a moderate negative relationship between temperature and SARS-CoV-2 transmissibility (r = −0.512, 95% CI: −0.680, −0.338, *p* < 0.001, I^2^ = 72.2%) with a non-significant predicted interval (95% CI: −1.18, 0.16) indicates that while most studies show a negative relationship between temperature and SARS-CoV-2 transmissibility, a future research might find no effect or even a positive effect, due to heterogeneity across studies (Figure 2). Similarly, the meta-analysis of regression coefficients indicates that SARS-CoV-2 transmissibility decreases by 0.02 (95% CI: −0.030, −0.020, *p* < 0.001, I^2^ = 0%) per degree Celsius increase in temperature, with no predicted interval (95% CI: −0.08, −0.01), indicating consistent effects across future studies (Figure 3).

**Figure 4:**
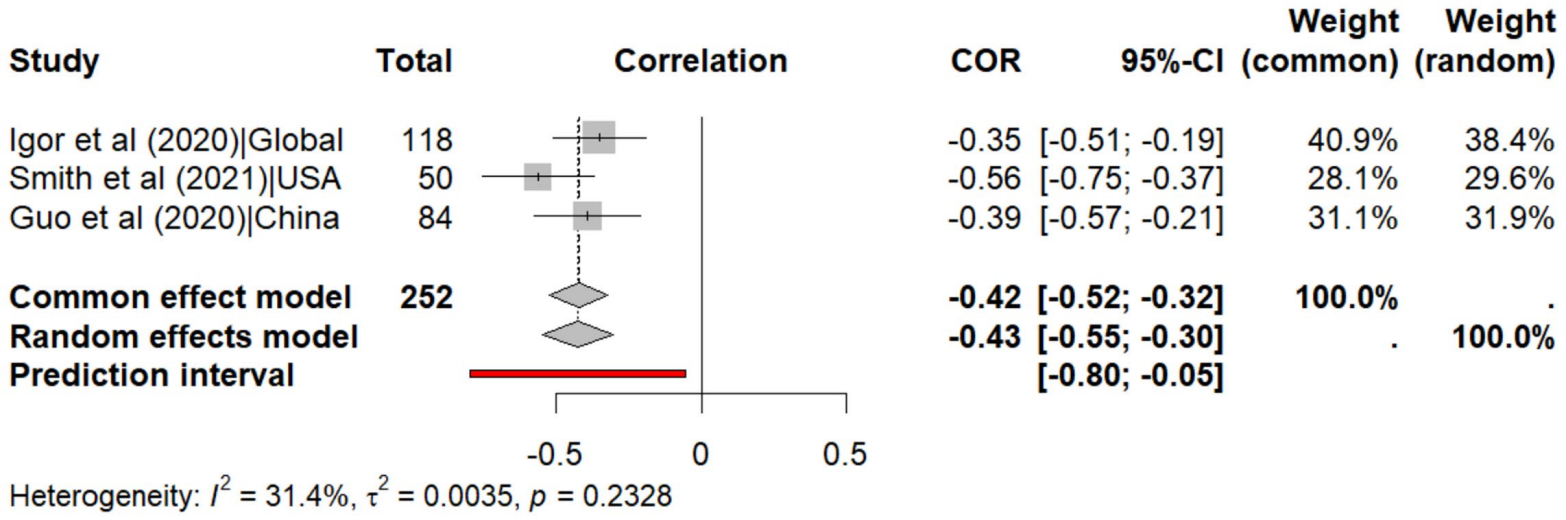
Meta-analysis of the correlation coefficients for humidity on explaining the transmissibility of SARS-CoV-2.

**Figure 5:**
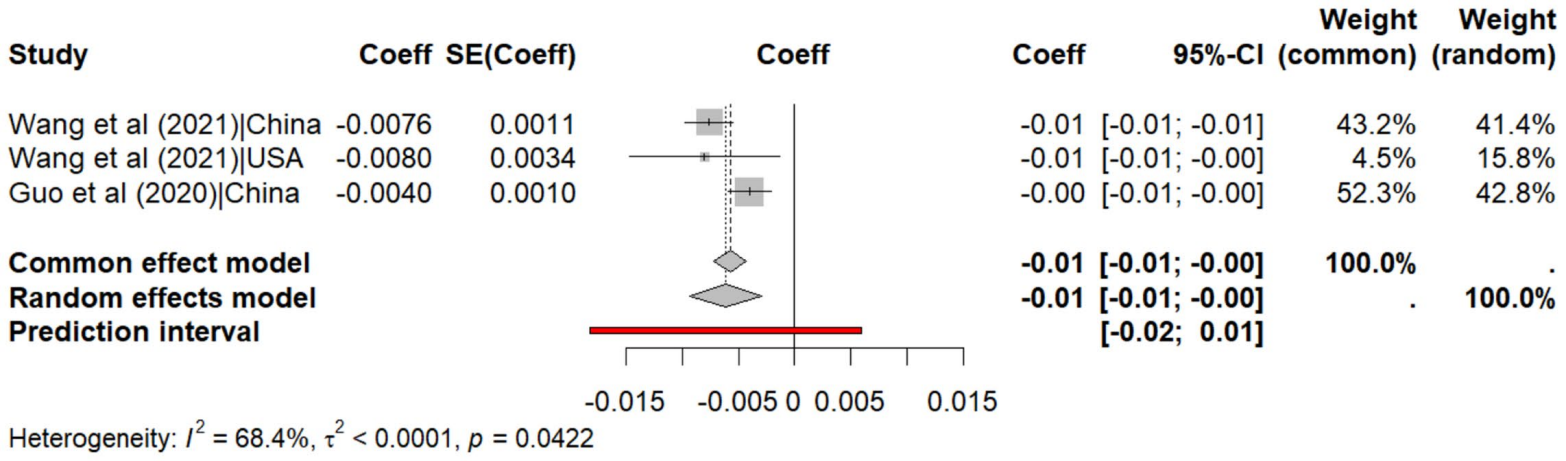
Meta-analysis of the effect size of regression coefficients for humidity on explaining the transmissibility of SARS-CoV-2.

The analysis of humidity reveals a weak negative correlation with transmissibility (r = −0.426, 95% CI: −0.547, −0.303, *p* < 0.001, I^2^ = 31.4%), with a predictive interval (95% CI: −0.80, −0.05) indicating that future studies are also likely to observe a negative correlation between humidity and SARS-CoV-2 transmissibility (Figure 4). Likewise, the meta-analysis of regression coefficients shows that humidity reduces transmissibility by 0.006 (95% CI: −0.009 to −0.003, p < 0.001, I^2^ = 68.4%) with predictive interval (95% CI: −0.02, 0.01) indicating that future studies are less likely to observe a negative correlation between humidity and SARS-CoV-2 transmissibility due to heterogeneity across studies (Figure 5).

## 4 Discussion

This systematic review and meta-analysis examined the influence of temperature and humidity on SARS-CoV-2 transmissibility. Overall, the findings indicate that temperature has a consistent negative association with transmissibility in regression analyses, while correlation estimates show high variability and a wide predictive interval, reflecting uncertainty about future effects. For humidity, regression analyses demonstrate less consistent negative associations due to wide predictive intervals, but correlation findings suggest a significant predictive interval, implying the relationship may be more consistent in future studies.

### 4.1 The effect of temperature on the transmissibility of SARS-CoV-2

This study found the inverse relationship between temperature and transmissibility of SARS-CoV-2. In addition, the 70.4% heterogeneity observed in the data may be due to differences in study characteristics. To account for this heterogeneity, a random-effects model was used. Additionally, other specific causes of heterogeneity across studies could be explained by factors such as geographic regions with different climatic conditions, differences in populations recruited in the primary studies, and variation in methods used to measure exposures, namely humidity and temperature, or the outcome of SARS-CoV-2 transmissibility (primarily identifying new cases as opposed to quantifying spread which was done using R0). These factors could perhaps influence how strongly and in which direction temperature affects SARS-CoV-2 transmissibility, leading to heterogeneity across the studies. Studies that were not included in this review because they failed to meet inclusion criteria, particularly not reporting the regression and correlation coefficient, suggest a similar conclusion that higher temperature is associated with a decrease in SARS-CoV-2 transmissibility (35, 36). Also, an increase in environmental temperature may reduce viral stability, as highlighted in a systematic review of laboratory studies to assess the effect of temperature on the viability of SARS-CoV-2, which demonstrated decreased virus stability in warmer conditions (37).

Previous systematic reviews and meta-analyses on other respiratory diseases apart from COVID-19 demonstrate a similar relationship evidenced by increasing incidence of respiratory tract infections as temperature decreases (38–40). Another study found that temperature and relative humidity were significantly negatively associated with the effective reproductive number (R). However, the authors emphasized that demographics, socioeconomic status, healthcare access, and human mobility were also included in the regression models, highlighting that temperature alone cannot fully explain transmission dynamics (41). This suggests that although temperature remains a significant and strong predictor in the transmissibility of SARS-CoV-2, its interaction with other predictors such as humidity, social factors, and mobility cannot be ignored (42).

### 4.2 The effect of humidity on the transmissibility of SARS-CoV-2

Humidity is another factor that was an exposure of interest in this review, as well as in other studies, examining the spread of respiratory viruses, including SARS-CoV-2 (43). This review found an inverse relationship between humidity and SARS-CoV-2 transmissibility, similar to other studies (43, 44). This is because higher humidity affects the stability and size of respiratory droplets, making them settle more quickly out of the air (43). Conversely, low humidity conditions may favor the survival of airborne droplets and contribute to the infection of airborne diseases (45). These findings suggest that humidity plays a significant role in influencing the transmissibility of SARS-CoV-2, but its impact is likely modulated by factors such as temperature, ventilation, and human behavior (46), highlighting the need to consider humidity in public health strategies for controlling respiratory infections.

### 4.3 Seasonal variability and implications

The findings from this meta-analysis suggest that temperature and humidity are significant climatic determinants of SARS-CoV-2 transmissibility. However, seasonal changes that affect both temperature and humidity levels also coincide with behavioral changes in populations, such as spending more time indoors during colder months, which could increase transmission of the virus (47). This underscores the importance of considering a multifactorial approach when assessing the impact of environmental conditions on virus transmission, rather than focusing on temperature or humidity in isolation.

### 4.4 Public health implications

The overall result of the meta-analysis on the transmissibility of SARS-CoV-2 shows an inverse relationship with temperature and humidity. The results of this review could have significant implications for public health policies. The finding that environmental factors like temperature and humidity do play a role in the spread of SARS-CoV-2 implies that they should be taken into consideration when designing public health preventive strategies. This should be done through incorporating considerations of such weather parameters (humidity, temperature) in designing effective and comprehensive control strategies that include established measures such as vaccination, mask-wearing, and social distancing (48–50). At the policy level, the findings show the importance of incorporating and integrating climate parameters into disease surveillance systems, pandemic preparedness and public health policies development, leading to a resilient local and global health system.

### 4.5 Study limitations

This systematic review and meta-analysis has several limitations that should be considered when interpreting and applying the findings. First, the review focused on studies that specifically reported the basic reproduction number (R0), which may have led to the exclusion of studies that explored the impact of temperature and humidity on SARS-CoV-2 transmissibility using other measures of transmission. Second, despite the comprehensive search strategy, the meta-analysis included only four studies due to the stringent inclusion criteria, particularly the requirement for correlation and regression coefficients. This small number of studies might limit the generalizability of the meta-analysis results. Third, although a random-effects model was used to account for variability among studies, the high heterogeneity observed in the meta-analysis of the correlation coefficient for temperature (I^2^ = 70.4% and I^2^ = 68.4%) indicates substantial inconsistency in the effect estimates across studies, which may arise from differences in study populations, methods, or other unmeasured factors which might have influenced the relationship between temperature and SARS-CoV-2 transmissibility solely from random variation. However, it is important to note that the I^2^ statistic has limitations, especially in meta-analyses with a small number of studies, where it can be imprecise and overestimate heterogeneity. Fourth, this review did not assess publication bias due to the limited number of studies included in some analyses, which reduces the power and reliability of publication bias tests. Fifth, the review relied on the quality of the included studies. While a risk of bias assessment was conducted, variations in study quality, as indicated by the JBI critical appraisal, could have influenced the overall findings. Specifically, some studies had limitations in addressing confounding factors, which could affect the validity of their results and, consequently, their contribution to the study narrative synthesis and meta-analysis.

## 5 Conclusion and recommendation

This systematic review and meta-analysis examined the influence of temperature and humidity on SARS-CoV-2 transmissibility, focusing on correlation rather than causation. The findings suggest an inverse relationship between temperature or humidity and the transmissibility of SARS-CoV-2. Humidity plays a role in SARS-CoV-2 transmission, with higher levels potentially reducing droplet viability. Since the study was based on the basic reproductive number, other unmeasured factors including public health interventions such as vaccination, mask-wearing, quarantine, lockdown, and social distancing, as well as population morbidity, socioeconomic status, and air quality, could significantly influence the dynamics of SARS-CoV-2 transmission. Furthermore, the findings of this study serve as a foundation for future research, encouraging the use of more robust methodologies and improved control of confounding variables in future pandemics or similar respiratory diseases. Through this, national and global emergency preparedness teams would be more aware of the weather parameters’ contribution to the transmissibility of SARS-CoV-2 and other similar viruses in the future and inform effective public health strategies.

## Data Availability

The original contributions presented in the study are included in the article, further inquiries can be directed to the corresponding author.

## References

[1] Kumar A Singh R Kaur J Pandey S Sharma V Thakur L . Wuhan to world: the COVID-19 pandemic. Front Cell Infect Microbiol. (2021) 11:596201. doi: 10.3389/fcimb.2021.596201, PMID: 33859951 PMC8042280

[2] Cucinotta D Vanelli M. WHO declares COVID-19 a pandemic. Acta Bio Medica Atenei Parmensis. (2020) 91:157–60. doi: 10.23750/abm.v91i1.9397, PMID: 32191675 PMC7569573

[3] Carrasco-Escaff T Garreaud R Bozkurt D Jacques-Coper M Pauchard A. The key role of extreme weather and climate change in the occurrence of exceptional fire seasons in south-Central Chile. Weather Clim Extremes. (2024) 45:100716. doi: 10.1016/j.wace.2024.100716

[4] WHO WHO COVID-19 dashboard (2024).

[5] Sarker R Roknuzzaman A Hossain MJ Bhuiyan MA Islam MR. The WHO declares COVID-19 is no longer a public health emergency of international concern: benefits, challenges, and necessary precautions to come back to normal life. Int J Surg. (2023) 109:2851–2. doi: 10.1097/JS9.0000000000000513, PMID: 37222700 PMC10498846

[6] Brito AF Semenova E Dudas G Hassler GW Kalinich CC Kraemer MU . Global disparities in SARS-CoV-2 genomic surveillance. Nat Commun. (2022) 13:7003. doi: 10.1038/s41467-022-33713-y 36385137 PMC9667854

[7] Mercatelli D Giorgi FM. Geographic and genomic distribution of SARS-CoV-2 mutations. Front Microbiol. (2020) 11:1800. doi: 10.3389/fmicb.2020.01800, PMID: 32793182 PMC7387429

[8] Gomes BBM Ferreira NN Garibaldi PMM de Lima Dias CFS Silva LN Dos Santos MAAL . Impact of SARS-CoV-2 variants on COVID-19 symptomatology and severity during five waves. Heliyon. (2024) 10:e40113. doi: 10.1016/j.heliyon.2024.e40113, PMID: 39605810 PMC11600076

[9] Mwiinde AM Siankwilimba E Sakala M Banda F Michelo C. Climatic and environmental factors influencing COVID-19 transmission—an African perspective. Tropic Med Infect Dis. (2022) 7:433. doi: 10.3390/tropicalmed7120433, PMID: 36548688 PMC9785776

[10] Yang X-D Su X-Y Li H-L Ma R-F Qi F-J Cao Y-E. Impacts of socio-economic determinants, spatial distance and climate factors on the confirmed cases and deaths of COVID-19 in China. PLoS One. (2021) 16:e0255229. doi: 10.1371/journal.pone.0255229, PMID: 34314442 PMC8315531

[11] Chen S Prettner K Kuhn M Geldsetzer P Wang C Barnighausen T . Climate and the spread of COVID-19. Sci Rep. (2021) 11:9042. doi: 10.1038/s41598-021-87692-z, PMID: 33907202 PMC8079387

[12] Marazziti D Cianconi P Mucci F Foresi L Chiarantini I Della Vecchia A. Climate change, environment pollution, COVID-19 pandemic and mental health. Sci Total Environ. (2021) 773:145182. doi: 10.1016/j.scitotenv.2021.145182 33940721 PMC7825818

[13] Qi H Xiao S Shi R Ward MP Chen Y Tu W . COVID-19 transmission in mainland China is associated with temperature and humidity: a time-series analysis. Sci Total Environ. (2020) 728:138778. doi: 10.1016/j.scitotenv.2020.138778, PMID: 32335405 PMC7167225

[14] Haque SE Rahman M. Association between temperature, humidity, and COVID-19 outbreaks in Bangladesh. Environ Sci Pol. (2020) 114:253–5. doi: 10.1016/j.envsci.2020.08.012, PMID: 32863760 PMC7447231

[15] McClymont H Hu W. Weather variability and COVID-19 transmission: a review of recent research. Int J Environ Res Public Health. (2021) 18:396. doi: 10.3390/ijerph18020396, PMID: 33419216 PMC7825623

[16] D'Arienzo M Coniglio A. Assessment of the SARS-CoV-2 basic reproduction number, R0, based on the early phase of COVID-19 outbreak in Italy. Biosaf Health. (2020) 2:57–9. doi: 10.1016/j.bsheal.2020.03.004, PMID: 32835209 PMC7148916

[17] Harris JE. Timely epidemic monitoring in the presence of reporting delays: anticipating the COVID-19 surge in new York City, September 2020. BMC Public Health. (2022) 22:871. doi: 10.1186/s12889-022-13286-7, PMID: 35501734 PMC9058738

[18] Islam A Sayeed MA Rahman MK Zamil S Abedin J Saha O . Assessment of basic reproduction number (R0), spatial and temporal epidemiological determinants, and genetic characterization of SARS-CoV-2 in Bangladesh. Infect Genet Evol. (2021) 92:104884. doi: 10.1016/j.meegid.2021.104884, PMID: 33930563 PMC8078038

[19] Jewell NP Lewnard JA. On the use of the reproduction number for SARS-CoV-2: estimation, misinterpretations and relationships with other ecological measures. J R Stat Soc Ser A Stat Soc. (2022) 185:S16–27. doi: 10.1111/rssa.12860, PMID: 35942193 PMC9350332

[20] Rayyan-Team. “Rayyan Ai web platform.” Available online at: https://www.rayyan.ai/ (2025). (Accessed 18 July 2025)

[21] Cochrane. Consumers and Communication Group resources for authors. (2016).

[22] Aromataris E Lockwood C Porritt K Pilla B Jordan Z, (editors). JBI Manual for Evidence Synthesis. Adelaide, Australia: JBI (2024). Available from: https://synthesismanual.jbi.global

[23] Cochrane. Cochrane handbook for systematic reviews of interventions. London, UK: Wiley-Blackwell, A John Wiley & Sons, Ltd, Publication (2008).

[24] R-Core-Team “A language and environment for statistical computing. R Foundation for Statistical Computing, Vienna, Austria.” (2023). Available online at: https://www.r-project.org/ (Accessed September 08, 2025)

[25] Salom I Rodic A Milicevic O Zigic D Djordjevic M Djordjevic M. Effects of demographic and weather parameters on COVID-19 basic reproduction number. Front Ecol Evol. (2021) 8:617841. doi: 10.3389/fevo.2020.617841

[26] Guo X-J Zhang H Zeng Y-P. Transmissibility of COVID-19 in 11 major cities in China and its association with temperature and humidity in Beijing, Shanghai, Guangzhou, and Chengdu. Infect Dis Poverty. (2020) 9:1–13. doi: 10.1186/s40249-020-00708-0 32650838 PMC7348130

[27] Smith TP Flaxman S Gallinat AS Kinosian SP Stemkovski M Unwin HJT . Temperature and population density influence SARS-CoV-2 transmission in the absence of nonpharmaceutical interventions. Proc Natl Acad Sci. (2021) 118:e2019284118. doi: 10.1073/pnas.2019284118, PMID: 34103391 PMC8237566

[28] Wang J Tang K Feng K Lin X Lv W Chen K . Impact of temperature and relative humidity on the transmission of COVID-19: a modelling study in China and the United States. BMJ Open. (2021) 11:e043863. doi: 10.1136/bmjopen-2020-043863, PMID: 33597143 PMC7893211

[29] Si X Bambrick H Zhang Y Cheng J McClymont H Bonsall MB. Weather variability and transmissibility of COVID-19: a time series analysis based on effective reproductive number. Experimental Results. (2021) 2:e15. doi: 10.1017/exp.2021.4 34192228 PMC8007945

[30] Kong JD Tekwa EW Gignoux-Wolfsohn SA. Social, economic, and environmental factors influencing the basic reproduction number of COVID-19 across countries. PLoS One. (2021) 16:e0252373. doi: 10.1371/journal.pone.0252373, PMID: 34106993 PMC8189449

[31] Landier J Paireau J Rebaudet S Legendre E Lehot L Fontanet A . Cold and dry winter conditions are associated with greater SARS-CoV-2 transmission at regional level in western countries during the first epidemic wave. Sci Rep. (2021) 11:12756. doi: 10.1038/s41598-021-91798-9, PMID: 34140557 PMC8211690

[32] Ogunjo S Olaniyan O Olusegun C Kayode F Okoh D Jenkins G. The role of meteorological variables and aerosols in the transmission of COVID-19 during harmattan season. Geohealth. (2022) 6:e2021GH000521. doi: 10.1029/2021GH000521, PMID: 35229057 PMC8865058

[33] Pahuja S Madan M Mittal S Pandey RM Madan K Mohan A . Weather parameters and COVID-19: a correlational analysis. J Occup Environ Med. (2021) 63:69–73. doi: 10.1097/JOM.0000000000002082 33177471 PMC7773164

[34] Rasul A. Relationship between climatic variables and reproduction number (R0) of confirmed COVID-19 cases. (2021).

[35] Lin S Wei D Sun Y Chen K Yang L Liu B . Region-specific air pollutants and meteorological parameters influence COVID-19: a study from mainland China. Ecotoxicol Environ Saf. (2020) 204:111035. doi: 10.1016/j.ecoenv.2020.111035, PMID: 32768746 PMC7406240

[36] Yang H-Y Lee JKW. The impact of temperature on the risk of COVID-19: a multinational study. Int J Environ Res Public Health. (2021) 18:4052. doi: 10.3390/ijerph18084052, PMID: 33921381 PMC8068915

[37] Aboubakr HA Sharafeldin TA Goyal SM. Stability of SARS-CoV-2 and other coronaviruses in the environment and on common touch surfaces and the influence of climatic conditions: a review. Transbound Emerg Dis. (2021) 68:296–312. doi: 10.1111/tbed.13707, PMID: 32603505 PMC7361302

[38] Mourtzoukou EG Falagas ME. Exposure to cold and respiratory tract infections. Int J Tuberc Lung Dis. (2007) 11:938–43. 17705968

[39] Eccles R Wilkinson J. Exposure to cold and acute upper respiratory tract infection. Rhinology. (2015) 53:99–106. doi: 10.4193/Rhino14.239, PMID: 26030031

[40] Zhang Y Yuan L Zhang Y Zhang X Zheng M Kyaw MH. Burden of respiratory syncytial virus infections in China: systematic review and meta–analysis. J Glob Health. (2015) 5:020417. doi: 10.7189/jogh.05.020417, PMID: 26682049 PMC4676581

[41] Smit AJ Fitchett JM Engelbrecht FA Scholes RJ Dzhivhuho G Sweijd NA. Winter is coming: a southern hemisphere perspective of the environmental drivers of SARS-CoV-2 and the potential seasonality of COVID-19. Int J Environ Res Public Health. (2020) 17:5634. doi: 10.3390/ijerph17165634, PMID: 32764257 PMC7459895

[42] Pérez-Gilaberte JB Martín-Iranzo N Aguilera J Almenara-Blasco M de Gálvez MV Gilaberte Y. Correlation between UV index, temperature and humidity with respect to incidence and severity of COVID 19 in Spain. Int J Environ Res Public Health. (2023) 20:1973. doi: 10.3390/ijerph20031973, PMID: 36767340 PMC9915304

[43] Luo W Majumder MS Liu D Poirier C Mandl KD Lipsitch M . The role of absolute humidity on transmission rates of the COVID-19 outbreak. MedRxiv [Preprint]. (2020). 10.1038/s41598-020-74089-7 PMC7552413 33046802

[44] Djordjevic M Salom I Markovic S Rodic A Milicevic O Djordjevic M. Inferring the main drivers of SARS-CoV-2 global transmissibility by feature selection methods. Geohealth. (2021) 5:e2021GH000432. doi: 10.1029/2021GH000432, PMID: 34568708 PMC8448988

[45] Rubin D Huang J Fisher BT Gasparrini A Tam V Song L . Association of social distancing, population density, and temperature with the instantaneous reproduction number of SARS-CoV-2 in counties across the United States. JAMA Netw Open. (2020) 3:e2016099. doi: 10.1001/jamanetworkopen.2020.16099 32701162 PMC7378754

[46] Maciorowski D Sharma D Kunamneni A. Environmental factors and their role in the transmission of SARS-CoV-2. Biosaf Health. (2021) 3:235–7. doi: 10.1016/j.bsheal.2021.07.005 34401711 PMC8357490

[47] Nottmeyer LN Sera F. Influence of temperature, and of relative and absolute humidity on COVID-19 incidence in England-a multi-city time-series study. Environ Res. (2021) 196:110977. doi: 10.1016/j.envres.2021.110977, PMID: 33684415 PMC7935674

[48] Srivastava N Saxena SK, (2020). Prevention and Control Strategies for SARS-CoV-2 Infection. In: Saxena S, (eds). Coronavirus Disease 2019 (COVID-19). Medical Virology: From Pathogenesis to Disease Control. Singapore: Springer.

[49] Wahl A Gralinski LE Johnson CE Yao W Kovarova M Dinnon KH III . SARS-CoV-2 infection is effectively treated and prevented by EIDD-2801. Nature. (2021) 591:451–7. doi: 10.1038/s41586-021-03312-w, PMID: 33561864 PMC7979515

[50] Wang Z Fu Y Guo Z Li J Li J Cheng H . Transmission and prevention of SARS-CoV-2. Biochem Soc Trans. (2020) 48:2307–16. doi: 10.1042/BST20200693, PMID: 33084885

